# Interfacial Characterization of Ruthenium-Based Amphiphilic Photosensitizers

**DOI:** 10.1021/acs.langmuir.2c01391

**Published:** 2022-07-29

**Authors:** Yousra Timounay, Andrea Pannwitz, David M. Klein, Anne-Laure Biance, Marlene E. Hoefnagel, Indraneel Sen, Alain Cagna, Marie Le Merrer, Sylvestre Bonnet

**Affiliations:** † Leiden University, Leiden Institute of Chemistry, Einsteinweg 55, 2333 CC Leiden, The Netherlands; ‡ Universität Ulm, Institut für Anorganische Chemie I, Albert-Einstein-Allee 11, 89081 Ulm, Germany; § Université de Lyon, Université Claude Bernard Lyon 1, CNRS, Institut Lumière Matière, F-69622 Villeurbanne, France; ∥ Teclis Scientific, 22 Ch. Des Prés Secs, 69380 Civrieux d’Azergues, France; ⊥ Wasabi Innovations Ltd., Sofia Tech Park Incubator, 111B, Tsarigratsko Shose, Sofia 1784, Bulgaria

## Abstract

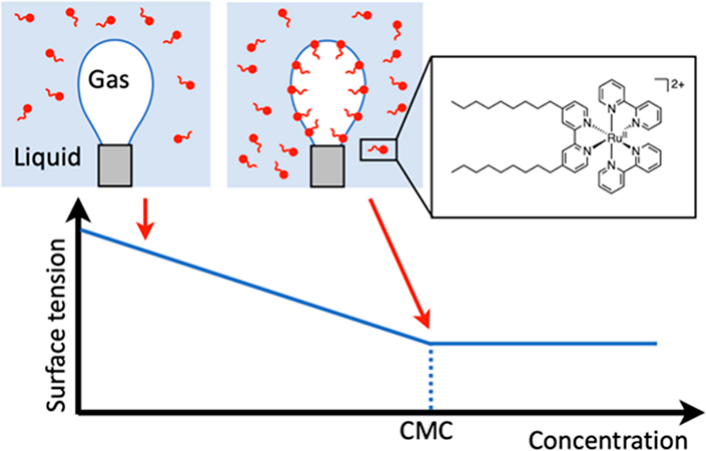

Nonreactive surfactant molecules have long been used and characterized for a wide range of applications in industries, life science, and everyday life. Recently, new types of functional amphiphilic molecules have emerged that bear another function, for example, a light-absorbing action, or catalytic properties. However, the surfactant properties of these molecules remain to date essentially unknown. In this context, we investigated here the interfacial activity of photocatalytic surfactants based on a ruthenium(II) tris-bipyridine core, functionalized with two alkyl tails. We realized a systematic characterization of the surfactant properties of these molecules at a water–air interface and studied the effect of the alkyl chain length and of the counterions (hexafluorophosphate or chloride) on these properties. Our data demonstrate that ruthenium surfactants with chloride counteranions form a denser layer at the interface, but their surfactant properties can dramatically deteriorate when the chain length of the alkyl tail increases, leading to simple hydrophobic molecules with poor surfactant properties for the longest chains (C17). These findings pave the way for a better use and understanding of photocatalytic soft interfaces.

## Introduction

Surfactants are a class of amphiphilic molecules that combine a polar, hydrophilic head and one or several apolar, lipophilic tail(s). Due to their amphiphilic character, these molecules often self-assemble in aqueous solutions, where they may also modify the surface tension of water–oil and water–gas interfaces, with a wide range of applications as cleaning agents, additives for flotation or extraction,^1^ food additives,^2^ antibacterial or anticancer drugs, or formulation additives for drug delivery.^3^ Surfactants represent an incredibly rich class of molecules, some of which include a metal center in the polar head.^4,5^ Recently, new molecules of that type have been introduced where the metal head provides a catalytic or light-harvesting function. For example, amphiphiles with light-absorbing or catalytic properties have been prepared to realize photocatalytic water oxidation or CO_2_ reduction at soft interfaces, with a perspective to produce solar fuels.^6,7^ Initially, these molecules have been designed to support the different components of a photocatalytic system onto liposomes.^6−12^ More recently, they have also been considered for the building of photocatalytic soap films and monolayers, because the escape of O_2_, H_2_, or CO_2_-reduction products such as CO or CH_4_ is easier at a water–gas interface than inside a liquid.^13^

In photocatalytic liposomes or monolayers at water–gas interfaces, a photosensitizer is needed to capture the solar light energy and start a photocatalytic process. Although amphiphilic molecules based on porphyrin architectures have been known for a long time,^14−16^ the by far most used amphiphilic photosensitizers nowadays are those based on a ruthenium(II) tris-bipyridine core, typically functionalized with one or two alkyl tails. Ruthenium tris-bipyridine is a very powerful photosensitizer for photocatalysis because it combines a high excited state energy (2.05 eV), a long excited state lifetime (∼1 μs), and a high oxidation potential (1.28 V vs NHE).^17^ Due to these exceptional photosensitizing properties, it can be used to trigger both water photooxidation and CO_2_ or proton photoreduction. Though the influence of alkyl chain functionalization on the photochemical and photophysical properties of ruthenium tris-bipyridine derivatives is well understood, the reverse effect, i.e., the effect of the ruthenium tris-bipyridine head on the properties of the surfactant, remains to date unknown. Most of the reported works using amphiphilic ruthenium tris-bipyridine conjugates have made use of different alkyl chain lengths without apparent rationale.^6,11,12^ Some of us very recently demonstrated, however, that the alkyl chain length has significant influence on the supramolecular properties and assembly of these molecules in lipid membranes, with dramatic consequences on photocatalysis.^7^ This study suggests that the surfactant properties of such molecules may also depend significantly on the chain length. Finally, ruthenium tris-bipyridine is a bicationic complex that bears counteranions, typically chlorides or hexafluorophosphates. Generally speaking, the influence of the counteranions on the solubility of nonamphiphilic ruthenium polypyridyl complexes is known: due to their excellent solvation in aqueous solution, chloride anions enhance the water solubility of cationic ruthenium polypyridyl complexes, compared to hexafluorophosphates anions.^17^ However, the influence of the counteranion on the interfacial and self-assembling properties of amphiphilic ruthenium compounds remains largely unexplored.

**Figure 1:**
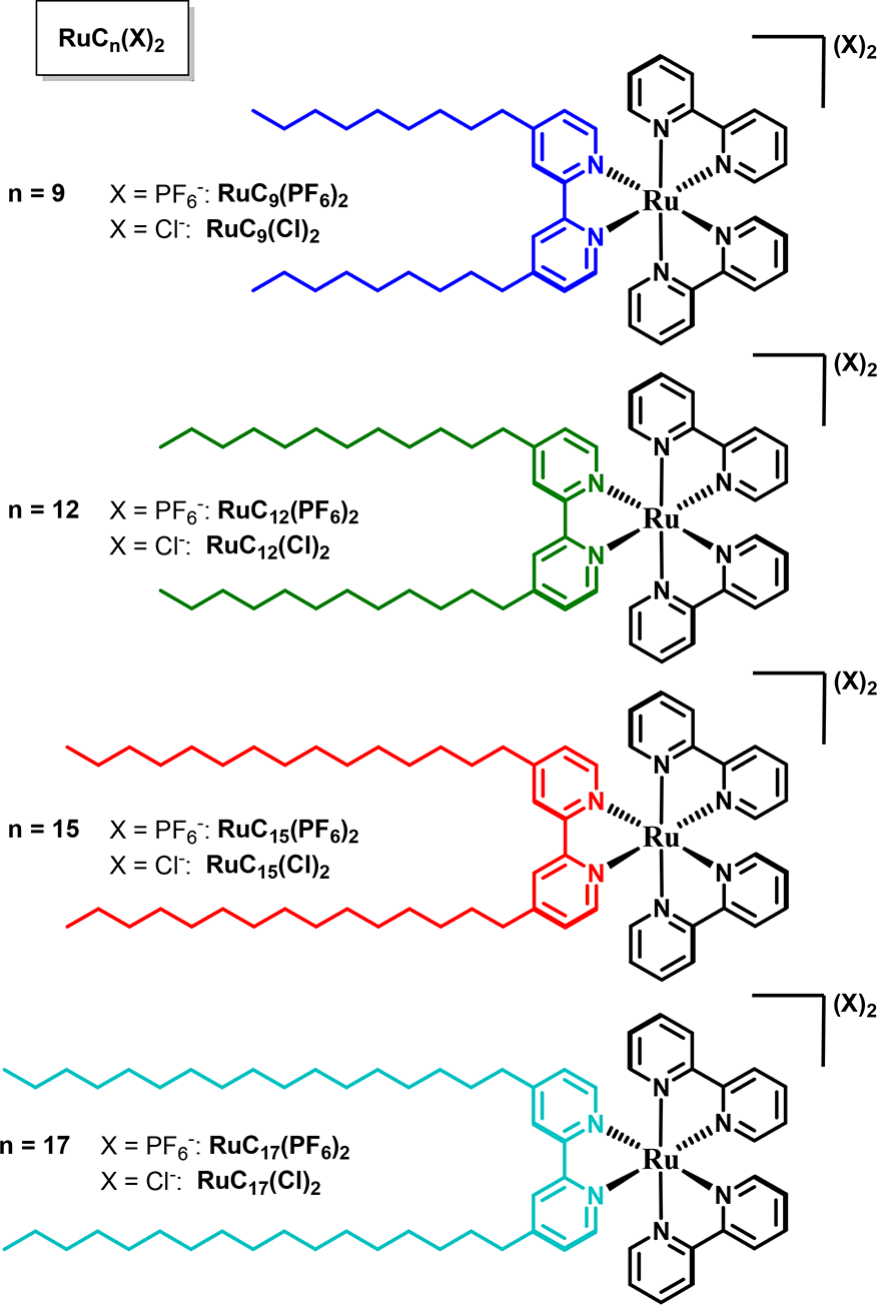
Overview of the investigated amphiphiles RuC_*n*_(X)_2_; the positively charged Ru(bpy)_2_^2+^ fragment is shown in black, and the color coding for the bipyridine ligand with different chain lengths corresponds to that used in other figures (blue for *n* = 9, green for *n* = 12, red for *n* = 15, and turquoise for *n* = 17). The counterion anion X^–^ is either PF_6_^–^ or Cl^–^.

**Figure 2:**
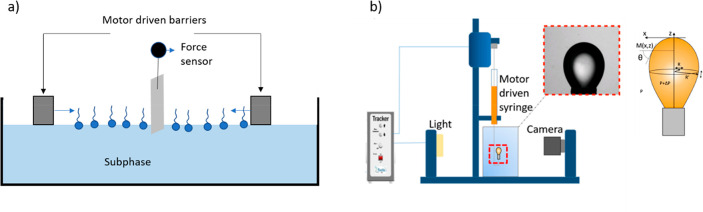
Principles of interfacial characterizations. (a) Scheme of a Langmuir–Blodgett trough. The surfactant layer is deposited on the subphase (blue molecules). The moving barriers compress the interfacial layer, and a force sensor measures the surface pressure. (b) Setup of the drop tensiometer: a gas (air) bubble is generated inside an aqueous solution, and its shape is observed via a camera. The interface lateral profile derives from the balance between surface tension and buoyancy, from which the surface tension value can be obtained.^20^

In this work, we investigated the influence of, on one hand, the alkyl chain length, and on the other hand, the nature of the counteranion, on the surfactant properties of bis-alkylated ruthenium tris-bipyridine compounds. To do so, we synthesized a series of amphiphilic complexes [Ru(bpy)_2_(C_*n*_bpy)](X)_2_ (hereafter called RuC_*n*_(X)_2_), where bpy is 2,2′-bipyridine, C_*n*_bpy is a series of 4,4′-dialkyl-2,2′-bipyridine where *n* = 9, 12, 15, and 17 is the number of carbons of the alkyl chains, and the counteranion X^–^ is either chloride Cl^–^ or hexafluorophosphate PF_6_^–^ (Figure 1). To understand the influence of the alkyl tail length on the ability of these molecules to modify the water–air interfacial properties, we performed different types of characterizations for both families of molecules: for the less soluble ones (PF_6_^–^ counterions), we used a Langmuir trough, and for the more hydrophilic ones (Cl^–^ counterions), we used a drop tensiometer (Figure 2). We characterized for the first time the compression isotherms and the adsorption kinetics of these compounds, which allowed us to characterize their area per molecule and to determine their surface pressure at the collapse, and the so-called interfacial Rosen parameters,^18,19^ which are commonly used to assess the performances of a surfactant. Based on our analysis, it was possible to identify which alkyl chain length and which counteranion lead to “real” surfactants, i.e., amphiphilic ruthenium molecules capable of modifying the water–air interface, as opposed to apparently amphiphilic molecules that are essentially hydrophobic and incapable of real surfactant behavior.

## Experimental Section

### Synthesis—General Methods

A Bruker AV300/1 FT-NMR spectrometer was used to record ^1^H NMR and ^13^C NMR as well as COSY, HSQC, and HMBC 2D spectra. Mestre Nova was used for the evaluation of the spectra. High-resolution mass spectrometry (HR-MS) was measured via direct injection on a mass spectrometer (Thermo Finnagan LTQ Orbitrap) with electrospray ionization. The ESI-MS mass spectra were measured with a ThermoFischer Scientific MSQ Plus electrospray ionization mass spectrometer with a 17–2000 *m*/*z* detection range and a resolution of approximately 0.5 *m*/*z*. Chromatographic silica columns were used for separating the components of the reaction mixtures, with a particle size of 40–63 μm and a surface area of 450–550 m^2^/g. The pore volume of the particles was 0.75–0.85 cm^3^/g. The silica powder was obtained from Screening Devices B.V. Dry loading was carried out by adsorbing the mixture onto either Celite or silica powder and solvent removal *in vacuo*, followed by deposition of the adsorbate on top of the column. The Celite was obtained from Sigma-Aldrich. To remove excess salts, size-exclusion chromatography (SEC) was performed using Sephadex LH-20 as a packing material and methanol as eluents. Sephadex LH-20 was purchased from VWR International B.V. Thin layer chromatography (TLC) was used as a first-hand method to test for the reaction mixtures’ compositions and the products’ purities. The TLC plates, composed of a fluorescent silica matrix with a pore volume of 0.75 cm^3^/g and a thickness of 0.2 mm, were supported on an aluminum sheet backing. The TLC plates were purchased from Supelco Analytical/Sigma-Aldrich. Elemental analysis was performed by Mikroanalytisches Laboratorium Kolbe in Oberhausen, Germany. The elemental content of the molecules was reported as the elements’ mass fraction percentage. Electronic absorption spectra were recorded on a Horiba Scientific Aqualog or on an Agilent Cary 50 Scan UV–vis spectrophotometer equipped with a single-cell Peltier temperature controller. Luminescence spectra were recorded on a Horiba Scientific Aqualog spectrophotometer equipped with a 150 W xenon lamp, an excitation range 230–620 nm, and an emission range 250–620 nm, using 3.5 mL 10 × 10 mm quartz cuvettes with four polished sides. Dynamic light scattering (DLS) was measured with a Zetasizer Nano-S from Malvern operating at 632.8 nm with a scattering angle of 173°. Structural data from single-crystal structures were retrieved from the Cambridge Crystal Structure Database,^21^ and distances between Ru atoms were analyzed with Mercury software.^22^

Melting points were measured on a Stuart melting point apparatus SMP30 with the temperature range of 20–400 °C. Temperature was increased from room temperature (RT) with an increase ramp of 2 °C/min in 0.4 °C increments until 180 °C and in 0.1 °C increments from 180 °C to the melting point.

All reagents for synthesis were commercially available. Dry and degassed solvents were purified by a solvent purification system. All reactions were carried out under an inert atmosphere, using standard Schlenk-techniques and N_2_ gas. Room temperature was typically between 20 and 25 °C. The synthesis of the ligands C_*n*_bpy was adapted from a previous study.^7,23^ The ligand 4,4′-dinonyl-2,2′-bipyridine (C_9_bpy) and the precursor complex *cis*-[Ru(bpy)_2_Cl_2_] were purchased from Sigma-Aldrich. The synthesis of the water-insoluble compounds RuC_*n*_(PF_6_)_2_ is reported elsewhere.^7^ The melting points of RuC_9_(PF_6_)_2_, RuC_12_(PF_6_)_2_, RuC_15_(PF_6_)_2_, and RuC_17_(PF_6_)_2_ were 210, 210, 215, and 217 °C, respectively.

#### RuC_9_(Cl)_2_

A mixture of *cis*-[Ru(bpy)_2_Cl_2_] (152 mg, 0.314 mmol, 1.00 equiv) and 4,4′-dinonyl-2,2′-bipyridine (128 mg, 0.314 mmol, 1.00 equiv) in a 1:1 mixture of ethanol and water (20 mL) was degassed via N_2_ bubbling for 15 min and then heated at 110 °C for 2 days. The solvent was removed to dryness, and the remaining dry, red solid was triturated in acetone. Filtering and washing with acetone yielded the desired product as an orange powder (224 mg, 0.233 mmol, 74%). ^1^H NMR (400 MHz, MeOD): δ 8.72 (d, *J* = 8.2 Hz, 4H, bpy-3-CH, bpy-3′-CH), 8.64 (d, *J* = 1.9 Hz, 2H, C9bpy-3-CH, C9bpy-3′-CH), 8.13 (tt, *J* = 7.9, 1.6 Hz, 4H, bpy-4-CH, bpy-4′-CH), 7.87–7.77 (m, 4H, bpy-6-CH, bpy-6′-CH), 7.64 (d, *J* = 5.8 Hz, 2H, C9bpy-6-CH, C9bpy-6′-CH), 7.50 (dtd, *J* = 7.2, 5.7, 1.3 Hz, 4H, bpy-5-CH, bpy-5′-CH), 7.35 (dd, *J* = 5.8, 1.8 Hz, 2H, C9bpy-5-CH, C9bpy-5′-CH), 2.86 (t, 4H, α-CH_2_), 1.74 (p, *J* = 7.4 Hz, 4H, β-CH_2_), 1.47–1.16 (m, 24H, 12 × CH_2_), 0.88 (t, 6H, CH_3_). ^13^C NMR (101 MHz, MeOD): δ 158.64 (bpy-C_q_), 158.60 (bpy-C_q_), 158.20 (C17bpy-2-C_q_, C9bpy-2′-C_q_), 156.59 (C9bpy-4-C_q_, C9bpy-4′-C_q_), 152.64 (bpy-3-CH, bpy-3′-CH), 152.51 (C9bpy-6-CH, C9bpy-6′-CH), 151.86 (bpy-4-CH, bpy-4′-CH), 139.05 (bpy-5-CH, bpy-5′-CH), 128.96 (C9bpy-5-CH, C9bpy-5′-CH), 128.86 (bpy-5-CH, bpy-5′-CH), 125.71 (C9bpy-3-CH, C9bpy-3′-CH), 125.60 (bpy-3-CH, bpy-3′-CH), 36.26 (α-CH_2_), 33.02 (CH_2_), 31.38 (CH_2_), 30.59 (CH_2_), 30.46 (CH_2_), 30.44 (CH_2_), 30.40 (CH_2_), 23.73 (CH_2_), 14.44 (CH_3_). ESI-MS (MeOH) *m*/*z* (%): calcd for [C_34_H_24_N_6_O_8_Ru]^2+^, 411.2; found, 411.1. Anal. Calcd for C_48_H_60_Cl_2_N_6_Ru + 2H_2_O + 1CH_3_OH: C, 61.24; H, 7.13; N, 8.74. Found: C, 61.44; H, 6.87; N, 8.79. Melting point: 217 °C.

#### RuC_12_(Cl)_2_

A mixture of *cis*-[Ru(bpy)_2_Cl_2_] (0.20 g, 0.41 mmol, 1.00 equiv) and 4,4′-didodecyl-2,2′-bipyridine (0.20 g, 0.41 mmol, 1.00 equiv) was added to a deoxygenated water/ethanol/chloroform mixture (1:1:1, 30 mL) and refluxed under N_2_ for 3 days at 110 °C. The solvents were removed by rotary evaporation, and the crude product was chromatographed on silica gel eluting with first acetone followed by acetone/water/brine (8:4:1). After removal of the solvents by rotary evaporation, the solids were redissolved in water, extracted by chloroform, dried with MgSO_4_, and filtered. Evaporation of the chloroform yielded 185 mg of RuC_12_(Cl)_2_·2.5NaCl (71%, 0.189 mmol). Size-exclusion chromatography with methanol as eluents was performed to remove extra salts. ^1^H NMR (400 MHz, CD_3_OD): δ = 8.74 (dd, *J* = 8.1, 1.6 Hz, 4H, bpy-3-CH, bpy-3′-CH), 8.66 (d, *J* = 1.6 Hz, 2H, C12bpy-3-CH, C12bpy-3′-CH), 8.13 (tt, *J* = 8.1, 1.6 Hz, 4H, bpy-4-CH, bpy-4′-CH), 7.83 (dt, *J* = 5.7, 1.9 Hz, 4H, bpy-6-CH, bpy-6′-CH), 7.64 (d, *J* = 5.9 Hz, 2H, C12bpy-6-CH, C12bpy-6′-CH), 7.50 (dtd, *J* = 7.4, 5.9, 1.2 Hz, 4H, bpy-5-CH, bpy-5′-CH), 7.36 (dd, *J* = 5.9, 1.5 Hz, 2H, C12bpy-5-CH, C12bpy-5′-CH), 2.86 (t, *J* = 7.9 Hz, 4H, α-CH_2_), 1.74 (p, *J* = 7.8 Hz, 4H, β-CH_2_), 1.41–1.19 (m, 36H, 18 × CH_2_), 0.88 (t, *J* = 7.0 Hz, 6H). ^13^C NMR (101 MHz, CD_3_OD): δ = 158.60 (C_q_), 158.57 (C_q_), 158.16 (C_q_), 156.53 (C_q_), 152.62 (CH, bpy-6-CH), 152.49 (CH, bpy-6′-CH), 151.86 (CH, C12bpy-6-CH, C12bpy-6′-CH), 139.05 (CH, bpy-4-CH, bpy-4′-CH), 128.97 + 128.87 (CH, C12bpy-5-CH, C12bpy-5′-CH, bpy-5-CH, bpy-5′-CH), 125.74 + 125.63 (CH, C12bpy-3-CH, C12bpy-3′-CH, bpy-3-CH, bpy-3′-CH), 36.24 (α-CH_2_), 33.05 (CH_2_), 31.36 (CH_2_), 30.75 (CH_2_), 30.72 (CH_2_), 30.62 (CH_2_), 30.46 (CH_2_), 30.45 (CH_2_), 30.43 (CH_2_), 23.72 (CH_2_), 14.46 (CH_3_). HR-MS *m*/*z*: calcd for [C_54_H_72_N_6_Ru]^2+^, 453.243 28; found, 453.242 72. Anal. Calcd for C_54_H_72_N_6_Cl_2_Ru·2.5 NaCl: C, 57.74; H, 6.46; N, 7.48. Found: C, 58.04; H, 6.51; N, 7.51. Anal. Calcd for C_54_H_72_N_6_Cl_2_Ru + 0.5 H_2_O (after size-exclusion chromatography): C, 65.77; H, 7.46; N, 8.52. Found: C, 65.95; H, 7.37; N, 8.49. Melting point: 217 °C.

#### RuC_15_(Cl)_2_

A mixture of *cis*-[Ru(bpy)_2_Cl_2_] (0.21 g, 0.43 mmol, 1.00 equiv) and 4,4′-dipentadecyl-2,2′-bipyridine (0.25 g, 0.43 mmol, 1.00 equiv) was added to a deoxygenated water/ethanol/chloroform mixture (1:1:1, 30 mL) and refluxed under N_2_ for 4 days at 110 °C. The solvents were removed by rotary evaporation, and the crude product was chromatographed on silica gel eluting with acetone/water/brine (8:4:1). After removal of the solvents by rotary evaporation, the solids were redissolved in chloroform, and the white precipitates were filtered off. This process was repeated with methanol as the solvent to yield 258 mg of RuC_15_(Cl)_2_·*x*NaCl. To remove excess salt, the red solid was purified by size-exclusion chromatography using methanol as eluents. *R*_f_ = 0.1 [SiO_2_, acetone/water/brine (8:4:1)]. ^1^H NMR (400 MHz, CD_3_OD): δ = 8.73 (d, *J* = 8.2 Hz, 4H, bpy-3-CH, bpy-3′-CH), 8.64 (d, *J* = 1.9 Hz, 2H, C15bpy-3-CH, C15bpy-3′-CH), 8.13 (tt, *J* = 8.0, 1.5 Hz, 4H, bpy-4-CH, bpy-4′-CH), 7.82 (td, *J* = 5.7, 1.8 Hz, 4H, bpy-6-CH, bpy-6′-CH), 7.64 (d, *J* = 5.8 Hz, 2H, C15bpy-6-CH, C15bpy-6′-CH), 7.50 (dq, *J* = 7.0, 5.5, 1.3 Hz, 4H, bpy-5-CH, bpy-5′-CH), 7.35 (dd, *J* = 5.9, 1.7 Hz, 2H, C15bpy-5-CH, C15bpy-5′-CH), 2.85 (t, *J* = 7.8 Hz, 4H, α-CH_2_), 1.74 (q., *J* = 8.0 Hz, 4H, β-CH_2_), 1.28 (m, 48H, 24 × CH_2_), 0.89 (t, *J* = 6.6 Hz, 6H, CH_3_). ^13^C NMR (101 MHz, CD_3_OD): δ = 158.62 (C_q_), 158.58 (C_q_), 158.17 (C_q_), 156.55 (C_q_), 152.63 (CH, bpy-6-CH), 152.49 (CH, bpy-6′-CH), 151.87 (CH, C15bpy-6-CH, C15bpy-6′-CH), 139.05 (CH, bpy-4-CH, bpy-4′-CH), 128.87 (CH, C15bpy-5-CH, C15bpy-5′-CH, bpy-5-CH, bpy-5′-CH), 125.62 (CH, C15bpy-3-CH, C15bpy-3′-CH, bpy-3-CH, bpy-3′-CH), 36.24 (α-CH_2_), 33.07 (CH_2_), 31.35 (CH_2_), 30.79 (CH_2_), 30.75 (CH_2_), 30.72 (CH_2_), 30.61 (CH_2_), 30.47 (CH_2_), 30.44 (CH_2_), 30.41 (CH_2_), 23.74 (CH_2_), 14.46 (CH_3_). HR-MS *m*/*z* calculated for [C_60_H_84_N_6_Ru]^2+^: 495.290 32, found: 495.290 07. Anal. Calcd for C_60_H_84_N_6_Cl_2_Ru·*x*NaCl: C, 67.90; H, 7.98; N, 7.92. Found: C, 44.76; H, 7.40; N, 5.12. Anal. Calcd for C_60_H_84_N_6_Cl_2_Ru + 1 H_2_O (after size-exclusion chromatography): C, 66.77; H, 8.03; N, 7.79. Found: C, 66.98; H, 8.12; N, 7.79. Melting point: 223 °C.

#### RuC_17_(Cl)_2_

A mixture of *cis*-[Ru(bpy)_2_Cl_2_] (754 mg, 1.56 mmol, 1.00 equiv) and 4,4′-diheptadecyl-2,2′-bipyridine (978 mg, 1.54 mmol, 0.99 equiv) in a 1:1:1 mixture of ethanol, water, and chloroform (60 mL) was degassed via N_2_ bubbling for 15 min and then heated at 110 °C for 2 days. After cooling to room temperature, the solvent was removed *in vacuo*. The reaction mixture was subjected to column chromatography (SiO_2_, acetone → 8:4:1 acetone/water/brine → 100:10:1 acetone/water/sat. KNO_3[aq]_) to isolate the red-orange fraction. The organic solvent was removed *in vacuo*, and the red compound was extracted with chloroform (3×). The combined organic layers were dried with MgSO_4_, and the solvent was removed to dryness. The red solid was taken up in methanol and subjected to an ion exchange column with Amberlite (50 g, presoaked with brine and washed 10 times with water and 3 times with methanol). The solvent was removed, and the red solid was taken up in a mixture of chloroform and 1:1 water/brine. The phases were separated, and the aqueous phase was extracted with chloroform (2×). The combined organic layers were dried with MgSO_4_, and the solvent was evaporated *in vacuo*. Trituration of the solid in acetone (100 mL) followed by removal of 50 mL of acetone at the rotary evaporator, cooling to room temperature, filtration, and washing with acetone (50 mL) yielded the desired compound as a chloride salt: RuC_17_(Cl)_2_·NaCl·3H_2_O) (1.14 g, 0.927 mmol, 60%). Size-exclusion chromatography with methanol as eluents was performed to remove extra salts. ^1^H NMR (400 MHz, MeOD): δ 8.67 (d, *J* = 8.2 Hz, 4H, bpy-3-CH, bpy-3′-CH), 8.59 (d, *J* = 1.9 Hz, 2H, C17bpy-3-CH, C17bpy-3′-CH), 8.15–8.03 (m, 4H, bpy-4-CH, bpy-4′-CH), 7.88–7.67 (m, 4H, bpy-6-CH, bpy-6′-CH), 7.61 (d, *J* = 5.8 Hz, 2H, C17bpy-6-CH, C17bpy-6′-CH), 7.46 (dtd, *J* = 7.2, 5.7, 1.3 Hz, 4H, bpy-5-CH, bpy-5′-CH), 7.31 (dd, *J* = 5.9, 1.8 Hz, 2H, C17bpy-5-CH, C17bpy-5′-CH), 2.82 (t, *J* = 7.9 Hz, 4H, α-CH_2_), 1.71 (p, *J* = 7.3 Hz, 4H, β-CH_2_), 1.44–1.11 (m, 56H, CH_2_), 0.87 (t, *J* = 6.6 Hz, 6H, CH_3_). ^13^C NMR (101 MHz, MeOD): δ 158.63 (bpy-C_q_), 158.60 (bpy-C_q_), 158.19 (C17bpy-2-C_q_, C17bpy-2′-C_q_), 156.56 (C17bpy-4-C_q_, C17bpy-4′-C_q_), 152.65 (bpy-6-CH), 152.52 (bpy-6′-CH), 151.87 (C17bpy-6-CH, C17bpy-6′-CH), 139.01 (bpy-4-CH, bpy-4′-CH), 128.95 (C17bpy-5-CH, C17bpy-5′-CH), 128.84 (bpy-5-CH, bpy-5′-CH), 125.67 (C17bpy-3-CH, C17bpy-3′-CH), 125.49 (bpy-3-CH, bpy-3′-CH), 36.26 (α-CH_2_), 33.09 (CH_2_), 31.35 (CH_2_), 30.80 (CH_2_), 30.77 (CH_2_), 30.74 (CH_2_), 30.63 (CH_2_), 30.49 (CH_2_), 30.44 (CH_2_), 30.42 (CH_2_), 23.75 (CH_2_), 14.47 (CH_3_). ESI-MS (MeOH) *m*/*z*: calcd for [C_64_H_92_N_6_Ru]^2+^, 523.32; found, 523.0. Anal. Calcd for C_64_H_92_Cl_2_N_6_Ru·NaCl·3H_2_O: C, 62.50; H, 8.03; N, 6.83. Found: C, 62.63; H, 8.03; N, 6.70. Anal. Calcd for C_64_H_92_Cl_2_N_6_Ru (after size-exclusion chromatography): C, 68.79; H, 8.30; N, 7.52. Found: C, 68.54; H, 8.55; N, 7.59. UV–vis *λ*_max_, nm (ε in M^–1^ cm^–1^): 453 (1.69 × 10^4^) in CH_3_CN. Emission, *λ*_max_: 615 nm in CH_3_CN. Melting point: 218 °C.

### Interfacial Characterization of the Compounds with PF_6_^–^ Counterions

To probe the interfacial properties of the non-water-soluble RuC_*n*_(PF_6_)_2_ compounds, Langmuir–Blodgett (LB) trough experiments were performed at room temperature (between 20 and 25 °C) using a commercial instrument (NIMA 601M). LB experiments consist in depositing a surface-active compound on a subphase (here, water) and compressing the deposited layer using two controlled moving barriers placed at the liquid–gas interface^24^ (Figure 2a). The surface pressure for different compressions is measured thanks to a vertical paper plunged in the solution and attached to a force sensor. In practice, solutions of RuC_*n*_(PF_6_)_2_ with *n* = 9, 12, 15, and 17 at 1 g/L in chloroform (HPLC grade, assay >99.8%) were prepared. The first step consisted in filling the trough with 50 mL of distilled water and depositing droplets of the solution under study on the distilled water bath. During this first step, the barriers of the trough were completely open, and the surface area of the air–water interface was 84 cm^2^. The deposited solution was uniformly distributed on the air–water interface, and its total volume was between 5 and 10 μL. We checked that the precise deposited volume has a negligible influence on the measurements (Figure S1). After 10 min, necessary for the evaporation of the chloroform, the newly created RuC_*n*_(PF_6_)_2_ monolayer at the air–water interface was compressed, while the surface pressure Π was measured. The latter corresponds to the reduction of the surface tension and is defined as Π = γ_0_ – γ with γ and γ_0_ being the surface tension and the initial surface tension of the interface, respectively. For all of the experiments, the area between the barriers was decreased from 84 to 22 cm^2^ at a speed of 10 cm^2^/min. For each RuC_*n*_(PF_6_)_2_ compound, two consecutive compression–decompression cycles were performed to check if the monolayer underwent an irreversible transformation such as the desorption of molecules from the air–water interface or an irreversible reorganization of the molecules at the interface, which typically manifests in qualitative differences between the first and second cycle. The experiments were performed in the dark to avoid the exposure of the photoactive RuC_*n*_(PF_6_)_2_ compounds to light.

### Interfacial Characterization of the Compounds with Cl^–^ Counterions

Solutions of RuC_*n*_(Cl)_2_ with *n* = 9, 12, 15, and 17 in demineralized water (by Mieuxa) were prepared at different concentrations and stirred for a few hours (up to 4 h) to solubilize the photosensitizers. For RuC_17_(Cl)_2_, which is more hydrophobic and hence less water-soluble, the complete solubilization of the compound required heating the samples for 1 h at 50 °C. The samples were left to cool down at room temperature (between 20 and 25 °C) before their use. According to DLS measurements, this procedure dissolved all aggregates of RuC_17_(Cl)_2_ in solution at 1 mg/mL corresponding to around 0.9 mmol/L (Figures S2 and S3). No precipitation of the RuC_17_(Cl)_2_ compound was observed during the months following the heating procedure. In addition, we checked that the heating step did not modify our results for the more soluble compound RuC_12_(Cl)_2_: heated and nonheated samples showed the same interfacial behavior (Figure 5).

The interfacial properties of each compound at the air–water interface were then probed using an automatic drop tensiometer (Tracker by Teclis,^20^ see Figure 2b). This technique, known as “the pendant drop” method, consists in creating a millimetric rising gas bubble in an aqueous solution of the surfactant under study using a computer-controlled syringe. The self-assembly of the surfactant at the created gas–water interface modifies the surface tension of the interface, which—in combination with Archimedes’ force—pushes the bubble to the top, directly influencing the shape of the air bubble. By extracting the profile of the bubble using a camera and fitting the theoretical Young–Laplace equation to the experimental bubble profile, the value of the surface tension is determined. These experiments can also be performed as a function of time.^20^

Similarly to the LB experiments, precautions were taken to avoid the exposure of the RuC_*n*_(Cl)_2_ compounds to light: the solutions were stored in the dark, and the automatic drop tensiometer was modified by the addition of a high pass filter (630 nm) to the white light source, and an opaque tubing between the sample and the camera was added.

For the current study, the experiments for RuC_*n*_(Cl)_2_ with *n* = 9, 12, and 15 consisted in generating a rising air bubble in a RuC_*n*_(Cl)_2_ solution at a given concentration and monitoring the value of the surface tension at the air–water interface during 14 h or until an equilibrium value was reached. During these experiments, air bubbles were subjected to low-amplitude sinusoidal variations of their volume to characterize the viscoelastic response of the interfaces (see the SI). For RuC_17_(Cl)_2_, experiments at concentrations below 2.4 mmol/L were performed following the protocol described above. For higher concentrations, the experiments consisted in generating a pendant drop of the RuC_17_(Cl)_2_ solution in demineralized water at the tip of a capillary in an air environment. Similarly, the evolution of surface tension at the air–water interface was monitored for up to 14 h. Despite being slightly sensitive to disruptions and evaporation, this technique was used for high concentrations, because it required a smaller volume of the solution and hence less compound. All of experiments were performed at room temperature (between 20 and 25 °C) for which the surface tension of pure water is γ_0_ = 72.3 mN/m. Note that 14 h might not be long enough to reach dynamical equilibrium, especially for the most hydrophobic complexes as described in the Results and Discussion section.

## Results and Discussion

### Compounds with Hexafluorophosphate Counteranions

**Figure 3:**
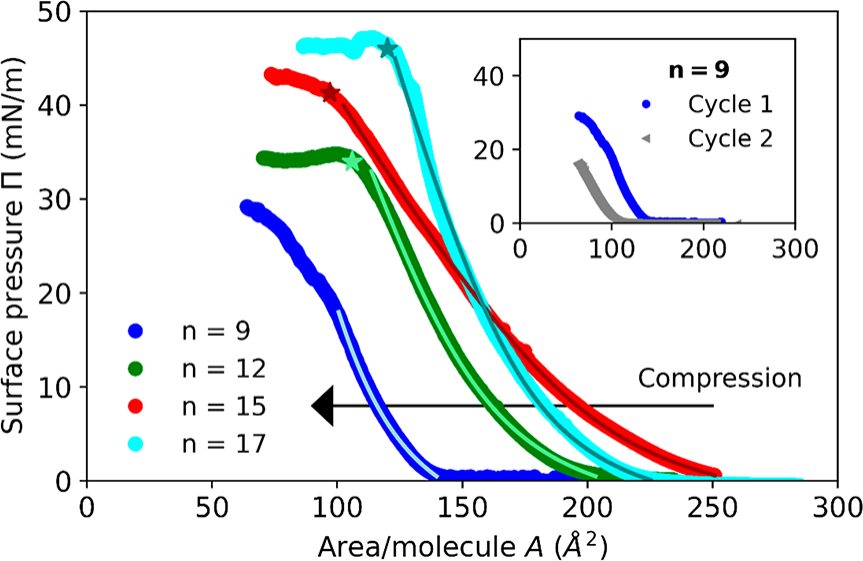
Surface pressure vs area per molecule upon compression of RuC_*n*_(PF_6_)_2_ monolayers during LB experiments. The legend shows the color coding for different *n* values. Lines correspond to fits with the modified Volmer model (eq 2 and Table 1). The inset shows a shift during the first two cycles for *n* = 9. The stars highlight the monolayer collapse.

The RuC_*n*_(PF_6_)_2_ compounds have a low water solubility, which made tensiometer measurements impossible. To characterize their monolayers at the air–water interface, Langmuir–Blodgett (LB) experiments were hence performed, as described in the Experimental Section and Figure 2a. The experimental surface pressure isotherms of all RuC_*n*_(PF_6_)_2_ compounds are presented in Figure 3. In contrast to RuC_9_(PF_6_)_2_, the more hydrophobic complexes RuC_12_(PF_6_)_2_, RuC_15_(PF_6_)_2_, and RuC_17_(PF_6_)_2_ underwent a collapse at high surface pressure (low surface tension) indicated by a plateau at the end of the compression phase. Langmuir monolayers undergo multiple phase changes during a compression (gaseous, liquid expanded, liquid condensed, and solid); here, the monolayer transitioned from a gaseous/liquid expanded phase (surface pressure values around zero) to a condensed state. The collapse occurred when the area per molecule reached a limiting value beyond which the monolayer cannot be compressed further without destabilizing its 2D nature and yielding structures in the third dimension, thereby leading to a slope change.^25^ The average values of the area per molecule, the surface pressure, and the tension at the collapse are reported in Table 1. RuC_9_(PF_6_)_2_ did not collapse, as observed by the missing plateau at low area per molecule. However, RuC_9_(PF_6_)_2_ showed lower pressure values during a second compression cycle (see the inset of Figure 3). This result suggests that the monolayer underwent an irreversible transformation during the first compression, even if a plateau was not observed. We hypothesize that desorption of RuC_9_(PF_6_)_2_ molecules from the interface into the bulk water phase occurred, which is compatible with the experimental observation that this short-tailed compound is slightly water-soluble. For *n* = 12, 15, and 17, the molecules are hydrophobic enough to remain at the interface during the compression of the monolayer; i.e., they behave as nonsoluble in water in the experimental conditions explored with the Langmuir trough. Furthermore, the surface pressure at the collapse (Π_col_) clearly increased with the length of the alkyl chain, ranging from 34.1 mN/m for RuC_12_(PF_6_)_2_ to 45.4 mN/m for RuC_17_(PF_6_)_2_ (Figure 3). This trend can be attributed to the increase of the hydrophobicity of these compounds with the length of the alkyl chain. Very hydrophobic molecules are more likely to remain at the air–water interface during compression, which would allow high surface pressure values to be reached.^26^

**Table 1:** LB Experiments for RuC_*n*_(PF_6_)_2_: Monolayer Properties at the Collapse (Average Values) and Parameter Fits for the Modified Volmer Model (Data of Figure 3)

	monolayer properties at the collapse			parameter fits for the modified Volmer model			
*n* (chain length)	surface pressure Π_col_ (mN/m)	surface tension γ_col_^a^ (mN/m)	area per molecule *A*_col_ (Å^2^)	α_0_ (Å^2^)	ε (m/N)	Π* (mN/m)	standard error of the estimate (Å^2^)
9				97	6.7	9.0	0.55
12	34.1 ± 0.4	38.2 ± 0.4	104 ± 1	140	8.3	6.1	1.35
15	41.3 ± 0.5	31.0 ± 0.5	98 ± 4	180	12	5.2	0.71
17	45 ± 1	27.3 ± 1	118 ± 2	160	6.3	6.2	1.27

a γ = γ_0_ – Π with γ_0_ = 72.3 mN/m

To further characterize the RuC_*n*_(PF_6_)_2_ monolayers at the air–water interface, the compression isotherms in the condensed state^27^ were fitted with a modified Volmer model that considers the molecules as hard disks with no long-range interactions and includes the expression of the two-dimensional monolayer compressibility coefficient ε (m/N).^28−30^ In this model, the excluded area per molecule α decreases when the surface pressure increases as described by the following equation:
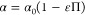
1with α_0_ (m^2^) being the excluded area per molecule in the gaseous/liquid expanded phase^27^ and Π (N/m) the surface pressure in the monolayer. In such a model, the modified Volmer equation can be written as follows:
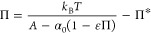
2where *k*_B_ = 1.38 × 10^–23^ J/K corresponds to the Boltzmann constant, *T* to the absolute temperature (K), *A* (m^2^) to the available area per molecule in the monolayer, and Π* to the cohesion pressure (N/m) accounting for the intermolecular interactions in the gaseous state (i.e., in the limit of low Π). Equation 2 provides an explicit expression for the area per molecule *A*(Π). Figure 3 shows that the experimental compression isotherms are well described by eq 2, at least for *n* = 12, 15, and 17; the corresponding fitting parameters are summarized in Table 1. As discussed previously, the compression isotherm for *n* = 9 suggests that a fraction of the molecules initially present in the monolayer desorbed during the applied compression; this desorption is likely at the origin of the deviation between the experimental data and eq 2 at high surface pressure values (Π > 18 mN/m).

According to this model, the area per molecule at the onset of the collapse, *A*_col_, did not vary significantly with the alkyl chain length, with an average value around 107 Å^2^ per molecule. If the tails are oriented perpendicular to the interface, the area per molecule at the collapse is a signature of the headgroup size, which is approximately the same for all investigated RuC_*n*_^2+^ molecules. Using the approximation of spherical headgroups organized in a hexagonal close packing, this *A*_col_ value translates into an average diameter of 11 Å for each headgroup, which includes their associated counterions and solvent molecules. This size is comparable with the intermolecular Ru···Ru distances in published solid state crystal structures of RuC_0_(PF_6_)_2_ compounds (CCDC 101676, 1115194, 1914096, 1115193, 101675, 1852899, and 1115195 with minimal Ru···Ru distances of *d* = 8.15–13.3 Å),^21^ which indicates a dominant influence of the headgroup–PF_6_^–^ ion pair on the average diameter per molecule and *A*_col_ at the water–air interface. We therefore conclude that the character and geometry of the contact ion pair of the ruthenium centered headgroup and the PF_6_^–^ counterions are similar at the air–water interface as in the single-crystalline solid state. The fitted values of α_0_, ε, and Π* did not vary appreciably with the tail length *n* (between 12 and 17), while it plays a crucial role in tuning the hydrophobicity of the RuC_*n*_(PF_6_)_2_ molecules. Similarly to *A*_col_, the average values of α_0_, ε, and Π* are probably a result of the properties of the headgroup, which is the same for all compounds. Finally, for *n* > 9, the excluded area per molecule α_0_, which corresponds to collapse for noncompressible monolayers, was larger than the area per molecule at the collapse *A*_col_, which confirmed the compressibility of these monolayers.

### Compounds with Chloride Counteranions

**Figure 4:**
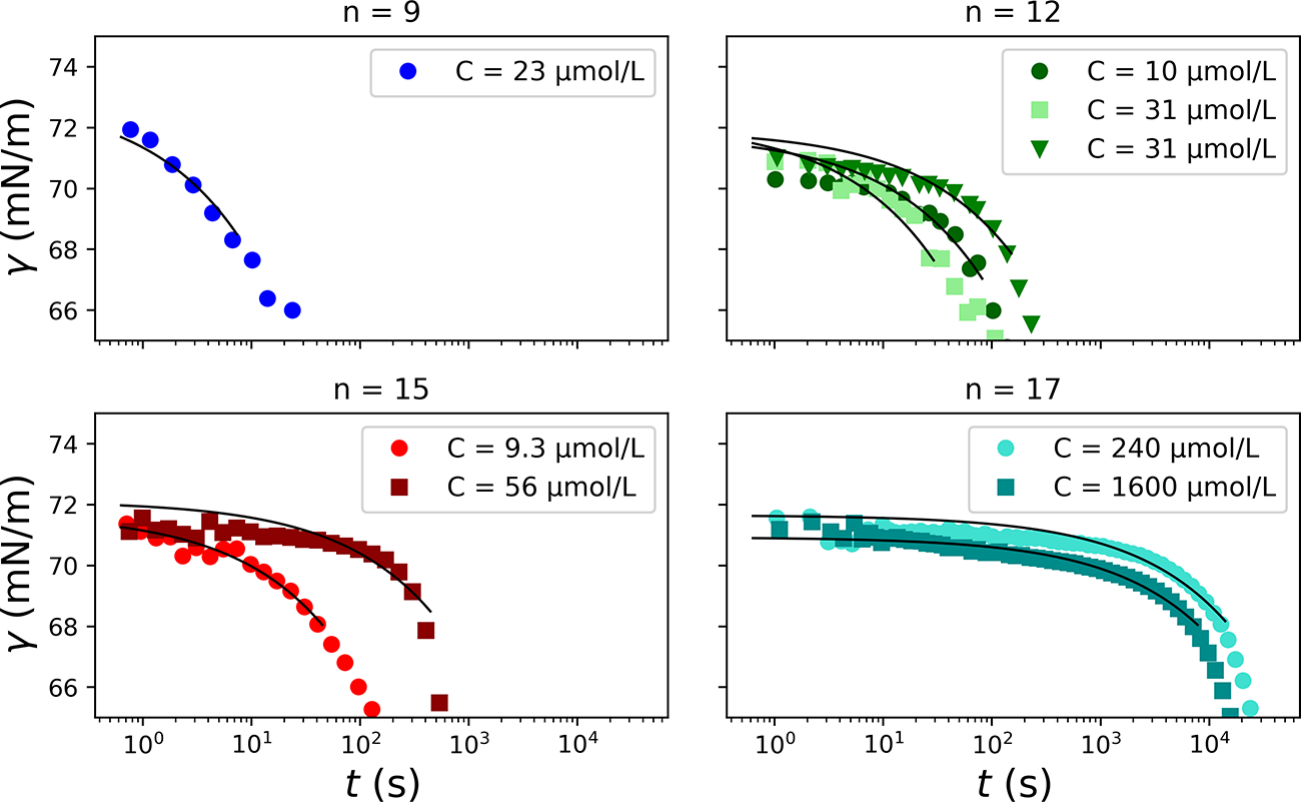
Surface tension γ as a function of time *t* for RuC_*n*_(Cl)_2_ samples obtained at various chain lengths and concentrations. The solid lines correspond to the Ward and Tordai equation in the short time approximation (eq 4) fitted to the experimental data for γ ≳ 65 mN/m. For clarity, the experimental data are shown as averaged over logarithmically spaced windows.

Qualitatively, the RuC_*n*_(Cl)_2_ compounds were found much more soluble than their hexafluorophosphate analogues, and at the highest concentrations used for this study, i.e., 5.6 × 10^–4^, 10^–4^, and 5.6 × 10^–5^ mol/L, for *n* = 9, *n* = 12, and *n* = 15, respectively, no aggregates nor precipitates were observed. The higher water solubility of the RuC_*n*_(Cl)_2_ compounds allowed their study using a drop tensiometer (Figure 2b), as described in the Experimental Section. By varying bulk concentrations of RuC_*n*_(Cl)_2_ in the solution, time-dependent surface tension measurements showed that these molecules decreased the value of surface tension over time, which is a strong indication that they indeed act as surfactants by adsorbing at the air–solution interface (Figure 4).

To model these time-dependent data, the adsorption of surfactant molecules to the air–water interface can be seen as a two-step process.^31,32^ First, the molecules diffuse from the bulk to the interface due to the concentration gradient. Second, adsorption makes surfactant molecules migrate from the subsurface to the surface itself. If the rate of diffusion is much slower than that of adsorption, the whole process is controlled by diffusion. In this limit, Ward and Tordai integrated the diffusion equation and obtained the following equation:^33^
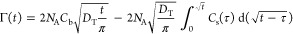
3where *t* (s) is the time, Γ(*t*) (m^–2^) is the surface excess (it corresponds to the inverse of the area per molecule *A* considered in Figure 3), *N*_*A*_ = 6 × 10^23^ mol^–1^ is the Avogadro number, *C*_b_ (mol/m^3^) is the bulk molar concentration, *D*_T_ (m^2^/s) is the diffusion coefficient, *C*_s_(τ) (mol/m^3^) is the subsurface concentration, and τ (s) is a variable of integration. If a surface-active monolayer behaves as an ideal surface at short times, an approximation can be obtained using the Henry adsorption isotherm for ionic surfactants γ_0_ – γ(*t*) = 2*k*_B_*TΓ*(*t*), which relates surface pressure to surface excess.^34^ We obtain a simple expression for the time dependence of the initial surface tension decrease at short times (eq 4):
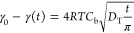
4where *R* = *N*_A_*k*_B_ is the molar gas constant. In practice, the apparent diffusion coefficient *D*_T_ of each RuC_*n*_(Cl)_2_ compound was obtained by fitting eq 4 to the adsorption curves γ(*t*) at the initial stage of adsorption (solid lines in Figure 4). This initial stage is defined here as γ ≳ 68 mN/m (γ_0_ – γ(*t*) ≲ 5 mN/m). The fitted diffusion coefficients for the different chain lengths and concentrations are reported in Table 2.

**Table 2:** Apparent Diffusion Coefficients *D*_T_ Deduced from the Adsorption Curves (Figure 4) at Various Tail Lengths and Concentrations of RuC_*n*_(Cl)_2_

chain length *n*	*C* (μmol/L)	*D*_T_ (m^2^/s)	*D*_T_/*D*_SE_	limiting kinetics
9	23	1.7 × 10^–10^	0.4	diffusion
12	10	8.4 × 10^–11^	0.2	diffusion
	31	2.4 × 10^–11^	0.06	adsorption
	31	3.7 × 10^–12^	0.009	adsorption
15	9.3	1.1 × 10^–10^	0.3	diffusion
	56	3.1 × 10^–13^	8 × 10^–4^	adsorption
17	240	4.9 × 10^–16^	1 × 10^–6^	adsorption
	1600	1.4 × 10^–17^	4 × 10^–8^	adsorption

These values should be compared to other estimations of the diffusion coefficient. For instance, the Stokes–Einstein equation^35^ can be used to calculate the Stokes–Einstein diffusion coefficients *D*_SE_ of RuC_*n*_(Cl)_2_ (eq 4), assuming that these molecules are spherical particles:
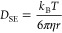
5where *k*_B_ = 1.38 × 10^–23^ J/K is the Boltzmann constant, *T* (K) is the absolute temperature, η (Pa s) is the dynamic viscosity of the solution containing the particle (water in our case), and *r* = 5.5 Å is the radius of the ruthenium head. With *T* = 293 K and η = 1.0016 mPa s, we find *D*_SE_ = 3.9 × 10^–10^ m^2^/s. This value is similar to the diffusion coefficient reported for the nonionic surfactant Triton-X-100 (2.9 × 10^–10^ m^2^/s) or the cationic surfactants C_14_TAB and C_16_TAB (4 × 10^–10^ m^2^/s).^36,37^ When *D*_T_ is comparable to *D*_SE_, this indicates that the kinetics are limited by diffusion, while *D*_T_ ≪ *D*_SE_ indicates the existence of an adsorption energy barrier (adsorption slower than diffusion). The last two columns of Table 2 show that the adsorption kinetics move from diffusion-limited to sorption-limited when the chain length *n* increases and when the concentration increases. This is consistent with literature results obtained for other cationic surfactants,^38,39^ although the adsorption time scales are much longer in our case. Indeed, for *n* = 17, this very slow adsorption implies that an equilibrium surface tension is never reached in our experiments that typically last 10–15 h. This shows the limited surfactant properties of the C17 compound. However, for *n* = 9, 12, and 15, equilibrium is reached after at most a few hours.

**Figure 5:**
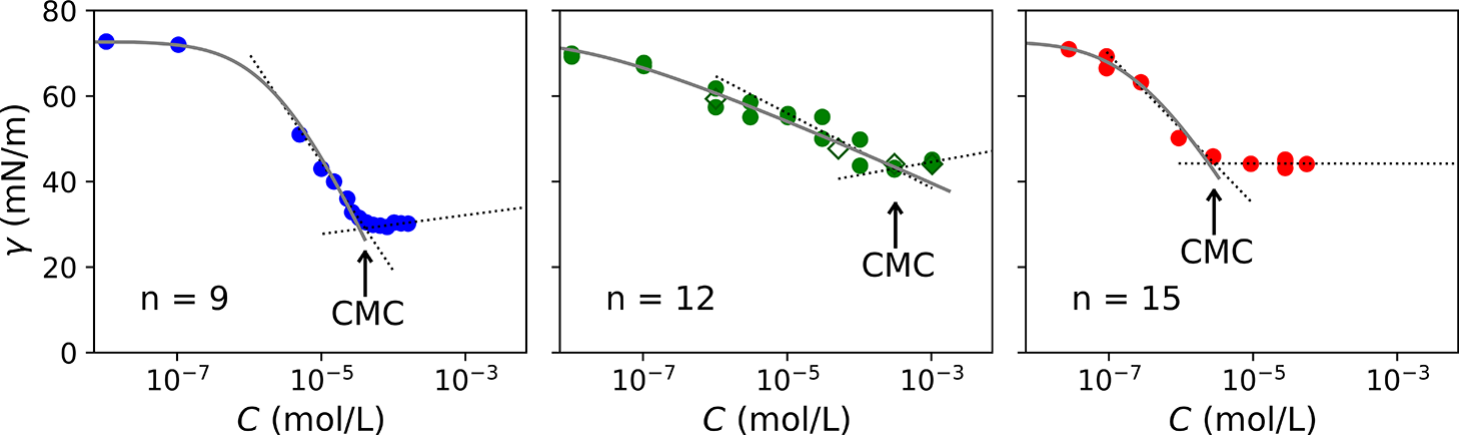
Plot of the equilibrium surface tension vs the concentration of RuC_*n*_(Cl)_2_ compounds with *n* = 9, 12, and 15 (dots). All plots include the fitting with the Volmer model (gray line) and the estimation of critical micellar concentration (arrow, deduced from the intersection of the dashed lines). For *n* = 12, we also show data obtained for heated samples (hollow diamonds).

The surface tension values obtained at the equilibrium for different compound concentrations are thus shown in Figure 5 for *n* = 9, 12, and 15. Equilibrium surface tension values first dropped with increasing concentration but became constant above a critical bulk concentration. This behavior is commonly observed for surface-active molecules when colloidal clusters, called micelles, are formed.^40^ Any surfactant molecule added after this point will go into the bulk and aggregate into micelles. The so-called critical micelle concentration (CMC) values are shown by an arrow in Figure 5 and are reported in Table 3. From these curves, one can evaluate the Rosen parameters, which are typically used to quantitatively assess the performances of a surfactant.^18,19^ The Rosen parameters of RuC_*n*_(Cl)_2_ compounds are defined as follows:

- The CMC value is the concentration at which surface-active molecules start forming micelles in polar solvents. In practice, micelle formation induces a break in the evolution of the equilibrium surface tension vs bulk concentration. These CMC values were determined by fitting the experimental data of Figure 5 before and after the slope change using logarithmic regressions. The CMC values of RuC_*n*_(Cl)_2_ compounds obtained from tensiometry experiments range between 2.9 and 320 μmol/L and depend strongly on the alkyl chain length, though no clear trend is observed. These values are lower by more than 1 order of magnitude than the CMC of common ionic surfactants (sodium dodecyl sulfate or SDS, cetyltrimethylammonium bromide or CTAB, 1–10 mM) but similar to one of the nonionic ones (hexaethylene glycol monododecyl ether or C_12_E_6_, 75 μM).^13,41,42^ This could be explained by the size of the molecules that tends to decrease the CMC. The effect of the hydrophobic chain length is more complex to interpret. Whereas the CMC is known to decrease with the alkyl chain length [as observed for the difference between RuC_9_(Cl)_2_ and RuC_15_(Cl)_2_],^41−43^ some specific packing and configuration emerges if the number of carbon is an odd or an even number.^44,45^ This may explain the higher CMC measured for *n* = 12, compared to *n* = 9 and 15. A similar behavior has indeed already been reported for other cationic surfactants.^46^
- The *C*_20_ value is the bulk concentration necessary to reduce the surface tension at the air–solvent interface by 20 mN/m.^19^ This parameter can be interpreted as the true efficiency of a molecule as a surfactant, as it characterizes its ability to adsorb at the interface. *C*_20_ values for RuC_*n*_(Cl)_2_ compounds are reported in Table 3. These values were strongly alkyl-chain dependent and followed more or less the CMC values (hence, with no clear trend).
- The *γ*_min_ parameter is the minimum value of surface tension reached in the surface tension measurement.^18^ It can be interpreted as the effectiveness of a surfactant, as it characterizes here its ability to reduce the surface tension, regardless of its concentration. The *γ*_min_ values for RuC_*n*_(Cl)_2_ ranged between 30 and 40 mN/m and increased with the chain length for *n* = 9, 12, and 15 (Table 3, Figure 5). In addition, these values are in the same order of magnitude as commonly used surfactants such as SDS or alkyltrimethylammonium bromide at the air–water interface.^18,41^

**Table 3:** List of the Surface-Related Parameters of All RuC_*n*_(Cl)_2_ Compounds

		Rosen parameters			parameter fits for Volmer model			
*n*	molecular mass from elemental analysis (g/mol)	CMC (μmol/L)	*γ*_min_ (mN/m)	*C*_20_ (μmol/L)	1/*K* (μmol/L)	α (Å^2^)	area/molecule at the CMC (Å^2^)	standard error of the estimate
9	961.09	41	29	5.0	2.3	19	28	0.14
12	977.18	320	43	31	0.010	120	230	0.74
15	1079.36	2.9	44	0.93	0.23	27	51	0.29
17	1117.45	n.d.^a^	n.d.	n.d.^a^	n.d.^a^	22^b^	n.d.^a^	n.d.^a^

a n.d.: not determined.

b This value has been deduced from the interfacial viscoelasticity data (see Figure S4 and the Supporting Information).

In terms of these Rosen parameters, RuC_9_(Cl)_2_ appeared as the most effective surfactant of the series, as it allowed reaching the smallest surface tension value, but RuC_15_(Cl)_2_ was the most efficient, because its CMC and *C*_20_ values are extremely low.

To further characterize the absorbed RuC_*n*_(Cl)_2_ monolayers at the air–water interface, we fitted our experimental data with the Volmer model for adsorption isotherms.^47^ Assuming a monomolecular adsorption, an adsorption isotherm relates the surfactant concentration in the bulk to the adsorbed amount at the interface. The Volmer model is derived assuming a finite molecular size, a nonlocalized adsorption, and only hard-core interactions between the adsorbed surfactants. For simplicity, we neglect the compressibility of the surfactant monolayer and the cohesion pressure considered for the insoluble PF_6_ compounds. The equation of state and the adsorption isotherm are given by eqs 6 and 7:
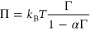
6
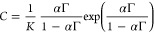
7where Π = γ_0_ – γ is the surface pressure (N/m), γ_0_ the initial surface tension of the interface, *K* an adsorption constant (L/mol), Γ the surfactant adsorption (m^–2^), α (m^2^) the excluded area, *T* the absolute temperature (K), k_B_ = 1.38 × 10^–23^ J/K the Boltzmann constant, and *C* the surfactant bulk concentration (mol/L). Equations 6 and 7 yield explicitly the bulk concentration *C* as a function of surface tension γ. We therefore fitted the experimental data *C*(γ) of Figure 5 using the Volmer model to deduce the best *K* and α values (reported in Table 3). As the concentration data are shown with a logarithmic scale, we fitted the logarithm values of the concentrations. The merit of the fit is thus characterized by the standard error of the estimate defined as  where *N* is the number of fitted data points. This model provides a good description of the experimental data as observed in Figure 5. The agreement is confirmed by looking at the viscoelastic properties of the interfaces (see the SI). The viscoelastic measurements also allowed us to estimate α for the C17 compound which did not reach equilibrium. Overall, we find that the excluded area α for the odd number of carbons (*n* = 9, 15, and 17), around 23 ± 4 Å^2^ (*d* = 5.4 ± 0.5 Å), is smaller than the Ru···Ru intermolecular distances found in published solid state crystal structures of Ru(bpy)_3_^2+^ with halide counterions, ranging between *d* = 7.5 and 7.8 Å (CCDC 1042836).^48^

### Comparison between Hexafluorophosphate and Chloride Counterions

**Figure 6:**
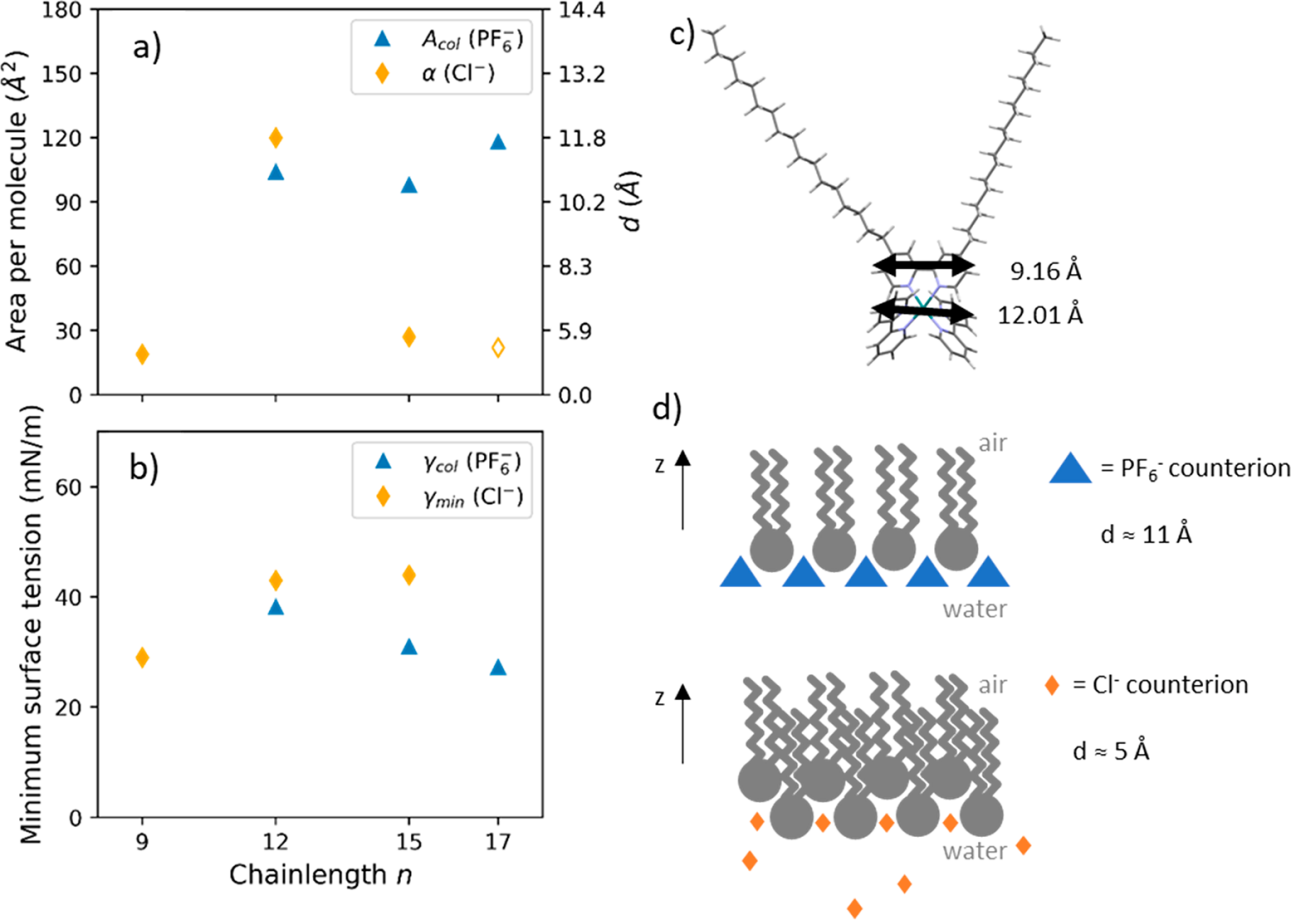
(a) Area per molecule and (b) minimum surface tension as a function of the length of the alkyl chain *n* for the two types of counterions. The area per molecule is the value at the collapse *A*_col_ for the RuC_*n*_(PF_6_)_2_ compounds (Table 1) and the excluded area α from the Volmer model for the RuC_*n*_(Cl)_2_ compounds (Table 3). The scale of corresponding intermolecular distance *d* is shown on the right axis. (b) The minimum surface tension corresponds to the surface tension at the collapse *γ*_col_ for the RuC_*n*_(PF_6_)_2_ compounds and to the minimum equilibrium surface tension *γ*_min_ for the RuC_*n*_(Cl)_2_ compounds. (c) DFT-minimized molecular geometry of RuC_17_^2+^ in the gas phase showing the dimensions of the ruthenium head. (d) Hypothesized arrangements of the bis-cationic amphiphiles at the air–water interface.

A comparison of the excluded area per molecule for the RuC_*n*_(Cl)_2_ compounds (α) to the area per molecule at the collapse for the RuC_*n*_(PF_6_)_2_ compounds (*A*_col_) is reported in Figure 6a. It shows that the compounds with chloride counterions would take up less area at the interface at full surface saturation. Even the area per molecule values at the CMC for the RuC_*n*_(Cl)_2_ compounds (estimated in Table 3) are smaller than the area per molecule at the collapse for the RuC_*n*_(PF_6_)_2_ compounds. Using the approximation of hexagonal close packing, we can translate the area per molecule into the intermolecular distance at the interface (Figure 6a, right). For the RuC_*n*_(PF_6_)_2_ compounds, it is comparable to the diameter of the ruthenium headgroup (around 10 Å^2^ estimated by DFT simulations; see Figure 6c and the Supporting Information for calculation details). However, for the RuC_*n*_(Cl)_2_ compounds with odd number of carbons, the diameter per molecule is significantly smaller. We therefore conclude that the RuC_*n*_(Cl)_2_ compounds tend to form a zigzag-type arrangement with respect to the interface-plane, and the RuC_*n*_(PF_6_)_2_ compounds form a uniform monolayer as drawn in Figure 6d. Another striking difference between the two types of counterions is the value of surface tension when the interface is saturated with surfactants, namely, γ_col_ for the RuC_*n*_(PF_6_)_2_ compounds and γ_min_ for the RuC_*n*_(Cl)_2_ ones. These values are compared and reported in Figure 6b. Even if these values seem comparable with the carbon chain corresponding to *n* = 12, the evolution of these parameters with *n* is intrinsically different; γ_min_ increases with *n* when γ_col_ decreases with *n*. This latter behavior can be linked with the hydrophobicity of the molecules that increases when the alkyl chain is longer.

## Conclusions

In conclusion, our first-in-kind study on the surfactant properties of the amphiphilic [Ru(bpy)_2_(C_*n*_bpy)]^2+^ complexes clearly demonstrates not only that the counterion type plays a major role on their solubility in water but also that it influences their aggregation and their molecular area at the air–water interface and, hence, their interfacial properties: hexafluorophosphate complexes take about 4 times as much area at the interface compared to chloride complexes. This effect suggests different assemblies at the interface, and we hypothesize a form of zigzag staggered arrangement of the chloride compounds, which probably does not take place for the hexafluorophosphate analogues.

Interestingly, the RuC_*n*_(PF_6_)_2_ series of compounds followed a clear trend: their surfactant properties are predominantly governed by the large contact ion pair formation at the interface and intermolecular van der Waals interactions of the tails. Their ability to lower the surface tension monotonously decreases with increasing alkyl chain length. On the contrary, the more water-soluble RuC_*n*_(Cl)_2_ compounds behave in a more contrasted way. In this series, changing the alkyl chain length of the molecule had a strong influence on its surfactant properties. While the C_9_ molecules did behave like good surfactants, the C_12_, C_15_, and C_17_ analogues were more hydrophobic (even with chloride anions) and therefore showed poorer surfactant properties; we may even claim that the surfactant properties of RuC_17_(Cl)_2_ are negligible. For the C_9_–C_15_ surfactants, as they are more compacted at the interface, some more complex interactions determine their properties, which depend not only on the length of the carbon tail but also probably on its conformation. To shed more light on this question, characterizations of RuC_*n*_(Cl)_2_ compounds with more and/or closer *n* values will be needed. Overall, depending on the targeted properties, a compromise between the surface activity of these amphiphilic ruthenium polypyridyl compounds and their ability to form very dense layers at the interface may be considered. If a good “soapy” surfactant is looked for, then RuC_*n*_(Cl)_2_ should be chosen, while on the contrary if a ruthenium compound that disturbs minimally the water–air interface is preferred, one of the PF_6_ complexes, or RuC_17_(Cl)_2_, should be chosen. Though still limited, notably regarding lower (*n* = 3–8) or even (*n* = 10, 14, 16) numbers of carbon atoms in each alkyl chain, these new findings represent an important step toward the preparation and understanding of photocatalytic soft interfaces.

## Supporting Information Available

Influence of initial coverage on Langmuir trough results; solubility of RuC_17_(Cl)_2_ at various temperatures; dilational viscoelasticity of the surface with RuC_*n*_(Cl)_2_; and geometry optimization of RuC_17_^2+^ via DFT (PDF)
