# Supplementary material for: Interfacial Characterization of Ruthenium-Based Amphiphilic Photosensitizers
Source: Langmuir. 2022 Jul 29;38(31):9697–707. doi: 10.1021/acs.langmuir.2c01391 (PMC9367009; doi:10.1021/acs.langmuir.2c01391)
Supplement: Supplementary file 1 — la2c01391_si_001.pdf [file la2c01391_si_001.pdf]

Interfacial characterization of ruthenium-based amphiphilic photosensitizers

Yousra Timounay,[d] Andrea Pannwitz,[a,b] David M. Klein,[a] Anne-Laure Biance,[c] Marlene E. Hoefnagel,[a] Indraneel Sen,[e] Alain Cagna,[e,*] Marie Le Merrer,[c,*] Sylvestre Bonnet[a,*]

[a] Leiden University, Leiden Institute of Chemistry, Einsteinweg 55, 2333 CC Leiden, The Netherlands; [b] Universität Ulm, Institut für Anorganische Chemie I, Albert-Einstein-Allee 11, 89081 Ulm, Germany; [c] Université de Lyon, Université Claude Bernard Lyon 1, CNRS, Institut Lumière Matière, F-69622, Villeurbanne, France; [d] Teclis Scientific, 22 Ch. Des Prés Secs - 69380, Civrieux d'Azergues, France; [e] Wasabi Innovations Ltd., Sofia Tech Park Incubator, 111B, Tsarigratsko Shose, Sofia 1784, Bulgaria.

*Corresponding authors: bonnet@chem.leidenuniv.nl, a.cagna@gmail.com, marie.le-merrer@univ-lyon1.fr

Supporting Information

1. TABLE OF CONTENT

1. Table of content 2
2. Influence of initial coverage on Langmuir trough results 3
3. Solubility of $RuC_{17}(Cl)_2$ at various temperatures 3
4. Dilational viscoelasticity of the surface with $RuC_n(Cl)_2$ 4
5. Geometry Optimization of RuC_{17}^{2+} via DFT 5
6. References 9

2. INFLUENCE OF INITIAL COVERAGE ON LANGMUIR TROUGH RESULTS

For the Langmuir trough measurements, the water-insoluble $RuC_n(PF_6)_2$ surfactants were dissolved in a chloroform solution. Figure S1 shows the surface pressure measurements for various volumes of deposited chloroform solution. We observe that the deposited volume has a negligible influence on the results.

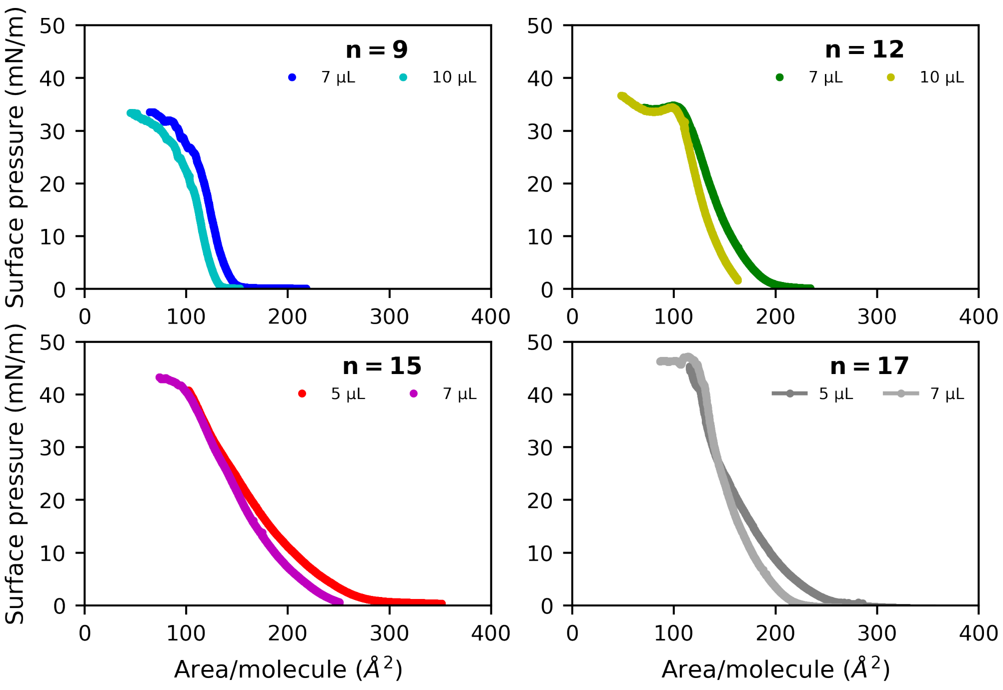

Figure S1. Surface pressure as a function of the area per molecule for $RuC_n(PF_6)_2$, for various volumes of chloroform solution deposited.

3. SOLUBILITY OF $RuC_{17}(Cl)_2$ AT VARIOUS TEMPERATURES

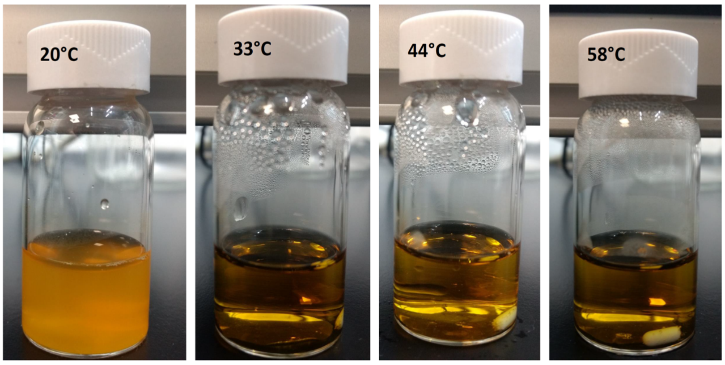

Figure S2. Photographs of $RuC_{17}(Cl)_2$ in water (concentration: 1 mg/mL = 0.9 mmol/L) at various temperatures showing the turbidity of the solution visually and the fully dissolved $RuC_{17}(Cl)_2$ at 55 °C, which is verified with DLS in Figure S3.

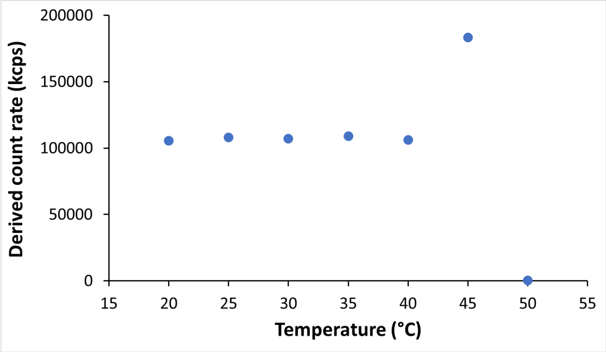

Figure S3. Dynamic light scattering data of $RuC_{17}(Cl)_2$ in water (1 mg/mL = 0.9 mmol/L) at various temperatures, showing that $RuC_{17}(Cl)_2$ is fully dissolved after heating up the solution. We hypothesize here that the compound is mostly insoluble at 40 °C and below, thus showing light scattering from large aggregates; at 45 °C it may start to solubilize, leading to more numerous and smaller aggregates that scatter light better, explaining the higher derived count rate; finally at 50 °C it becomes fully soluble, hence bringing the derived count rate close to zero.

4. DILATIONAL VISCOELASTICITY OF THE SURFACE WITH $RuC_N(Cl)_2$

In the drop tensiometer experiments, bubbles/drops were subjected to low-amplitude sinusoidal variations of their volume at a frequency of 0.2 Hz to characterize the viscoelastic response of the air/$RuC_n(Cl)_2$ solution interface.[1] The volume variations result in sinusoidal area variations of 8 – 12% of the initial surface area of the bubble or drop. By simultaneously tracking the evolution of surface tension as a function of time and controlling the surface area of the bubble/drop, the complex viscoelastic dilational modulus of the interface, defined as the ratio of the variations of surface tension over relative area variation can be extracted (see ref.[2] for more details).

Figure S4 shows the measured viscoelastic modulus E (the absolute value of the complex modulus) as a function of surface pressure $\Pi = \gamma_0 - \gamma$, where γ is the surface tension averaged over one period. Note that we also found (data not shown) that the imaginary part of the complex modulus (so-called loss modulus, characterizing the viscous response of the surface) was always much smaller than its real (so-called storage modulus, characterizing the elastic response of the surface), with a ratio between 0.05 and 0.13. This indicates a quasi-elastic behavior of the interface where the surfactant monolayer behaves as if it was insoluble on the timescale of an oscillation period.

We observe that E increases with the surface pressure Π as a result of the adsorption of the molecules at the interface.[3] This behavior is also expected from the Volmer adsorption model previously considered (Eqs. 6 and 7). If we neglect the surfactant adsorption/desorption during an oscillation, the viscoelastic modulus E is equal to the Gibbs-Marangoni modulus, which characterizes the variations of surface tension or surface pressure with relative changes of surfactant coverage, and can be deduced from Eq. 6:

$$E \approx \Gamma \frac{d\Pi}{d\Gamma} = k_B T \frac{\Gamma}{(1-\alpha\Gamma)^2} \qquad \textit{Eq. S1}$$

Using Eq. 6, this provides an explicit prediction for $E(\Pi)$ as shown in Figure S4, where the excluded area α is the value fitted on the equilibrium measurements $\gamma(C)$ of figure 6 for n = 9, 12, 15 (see table

3). We observe a reasonable agreement with the experimental data. For n = 17, no equilibrium data are available, so we simply fit the $E(\Pi)$ data with Eq. S1. The fitted value of the excluded area α is found to be 22 Å², close to the values found for n= 9 and 15.

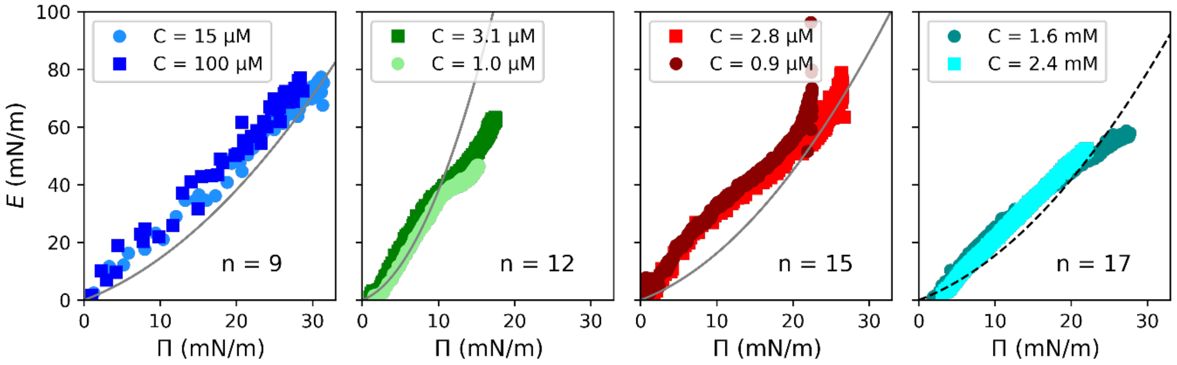

Figure S4. Viscoelastic modulus E as a function of surface pressure Π for $RuC_n(Cl)_2$. The curves show the prediction of the Volmer model, which was deduced from the fits of Figure 6 (grey lines for n = 9, 12, 15) or directly fitted on the elasticity data (dashed black line for n = 17).

5. Geometry Optimization of RuC_{17}^{2+} via DFT

Geometry optimization of RuC_{17}^{2+} without counterions in the gas phase was performed computationally using DFT with ADF2019 from SCM at the PBE0/TZP level in vacuum. The resulting 3D-geometry was analyzed with the program Mercury and is depicted in the main manuscript. The coordinates are reported in the table below.

Table S2. DFT-computed XYZ-coordinates of the optimized geometry of RuC_{17}^{2+} in the gas phase.

Atom	X	Y	Z
C	0.469540831	-1.275729996	3.747377319
C	-0.361724782	-0.361363354	4.40877888
C	-1.736885965	-0.309737229	4.154072851
N	-2.309545396	-1.150217595	3.234275989
C	-1.51206812	-2.025069493	2.575873906
C	-0.145593786	-2.119302006	2.806371301
Ru	-4.436559141	-0.955051392	2.986321666
N	-4.990581885	-2.236116686	4.612047832
C	-6.333282755	-2.29190484	4.894165475
C	-6.804172038	-3.059348533	5.968517345
C	-5.908844698	-3.774898157	6.760278998
C	-4.545427705	-3.712137309	6.463068527
C	-4.127399065	-2.933102722	5.388798545
C	-7.209660815	-1.505746165	4.003129151
N	-6.572062586	-0.767396882	3.036915559
C	-7.315171308	-0.030489189	2.177623104
C	-8.704495595	0.018335659	2.237112612
C	-9.363235227	-0.727082483	3.21762282
C	-8.607857089	-1.492605457	4.103041017

C	-2.665288402	0.606512377	4.852779323
N	-3.976821538	0.54357464	4.455849984
C	-4.877968923	1.342177084	5.075640946
C	-4.533055826	2.212224049	6.101651359
C	-3.200154748	2.288357412	6.540244189
C	-2.272378576	1.471745564	5.879532986
H	-6.763929	0.531679095	1.428241306
H	-5.380347533	-5.600418061	0.660152498
N	-4.083379821	0.223553159	1.232986367
H	-4.433296215	-2.341154734	-1.988031428
H	-5.007387344	-4.730556631	-1.678074213
H	-3.075657848	-2.852798691	5.127537691
H	-5.132250076	-4.021237996	2.57602373
H	-3.74472367	2.031128507	2.195846831
H	-3.415796165	2.096652675	-2.107181114
H	-3.308345207	3.313953901	0.099779116
H	-9.109915991	-2.08291251	4.864474078
H	-9.254272421	0.628829857	1.522702515
H	-1.232885667	1.497364273	6.195519502
H	-5.309519125	2.814542155	6.571610788
H	-7.866406068	-3.095970631	6.194340731
H	-3.809987799	-4.254244232	7.055188291
H	0.077506084	0.309618489	5.142231646
H	0.435878893	-2.858024497	2.255823712
H	-10.44990591	-0.71571671	3.290559832
H	-2.00418149	-2.672451763	1.854477727
H	-6.270610149	-4.371276855	7.59674477
H	-5.907641006	1.253567157	4.738040109
C	-4.140455302	-0.452247417	0.039064573
C	-4.4694874	-1.8886535	0.128860164
C	-4.588019157	-2.730422997	-0.985804459
C	-4.912497478	-4.074081222	-0.814100875
C	-5.117452857	-4.559353845	0.479528737
C	-4.981813771	-3.684896957	1.553515876
N	-4.658919885	-2.379097253	1.396764664
C	-3.901194747	0.21418749	-1.1702893
H	-3.946836731	-0.324126768	-2.112808789
C	-3.60165331	1.574786355	-1.169274408
C	-3.541991118	2.251692079	0.051012662
C	-3.785501448	1.544557187	1.224518525
C	-2.782603506	3.214023712	7.651891845
H	-3.577370633	3.247825155	8.413778139
H	-1.882112584	2.814294842	8.143180504
C	-2.494234714	4.659805289	7.154623855
H	-1.720948957	4.623507815	6.368238802
H	-3.405165645	5.068146917	6.685106071
C	-2.035550923	5.579653956	8.301055458
H	-2.802984674	5.578089648	9.094597567

H	-1.118899263	5.162781599	8.753528996
C	-1.768955444	7.029642739	7.854592334
H	-2.689549319	7.450754063	7.413221262
H	-1.007107613	7.033260875	7.054849531
C	-1.29925438	7.931800511	9.012201563
H	-2.057388323	7.91218916	9.814974004
H	-0.375889626	7.510341576	9.447388018
C	-1.04227975	9.391664447	8.594532178
H	-0.282800811	9.414230306	7.79270463
H	-1.966153525	9.814159173	8.160805732
C	-0.576605555	10.28133185	9.76273506
H	-1.335055167	10.25001153	10.56501247
H	0.348076003	9.858359396	10.19422635
C	-0.324257585	11.7467548	9.361936044
H	0.437112439	11.78019326	8.562305248
H	-1.248424418	12.1693659	8.928719644
C	0.134164706	12.63023987	10.5376649
H	1.058440649	12.20729115	10.97036559
H	19.95822155	7.720307304	-0.20698467
H	-0.6271296	12.59178051	11.33702052
C	1.952602011	-1.32093305	4.007361597
H	2.161191924	-0.922374021	5.011788209
H	2.2946341	-2.367964569	3.997042671
C	2.761513899	-0.507874789	2.954280661
H	2.591110916	-0.943090094	1.95544358
H	2.369213542	0.522880056	2.920380338
C	4.269864149	-0.478159429	3.266980165
H	4.42216131	-0.01777057	4.258403793
H	4.650046261	-1.511496986	3.34133831
C	5.08779792	0.29638601	2.21444412
H	4.668501947	1.312065209	2.10376133
H	4.977049114	-0.19562235	1.232268759
C	6.584633168	0.406937873	2.568103615
H	6.686138354	0.915629452	3.542903397
H	7.00637613	-0.604485931	2.700486241
C	7.405872903	1.170264077	1.510769848
H	7.344864549	0.635923325	0.546351523
H	6.949429702	2.162336556	1.345303045
C	8.885579716	1.359337911	1.899053669
H	8.938003363	1.903225621	2.858691126
H	9.347948905	0.372528742	2.076395292
C	9.702182564	2.12466048	0.839876665
H	9.688178576	1.560616934	-0.109176726
H	9.208722417	3.091101148	0.632907128
C	11.16214597	2.389117579	1.256914035
H	11.66331406	1.428225836	1.469290418
H	11.16882934	2.95731491	2.203692492
C	11.96967065	3.164078671	0.198860956

H	11.99865361	2.580126107	-0.737866462
H	11.44189405	4.104783729	-0.039588857
C	13.40897484	3.495510126	0.638492131
H	13.37389424	4.07976925	1.574909655
H	13.94358721	2.559387305	0.878312468
C	14.20788558	4.282723815	-0.418074841
H	14.27157473	3.686294386	-1.345333862
H	13.6536792	5.201690553	-0.679863579
C	15.62822772	4.664354626	0.040237599
H	16.18698359	3.747696734	0.29970999
H	15.56076999	5.25819462	0.968953082
C	16.42043573	5.463308813	-1.012602275
H	16.504724	4.864550318	-1.936931367
H	15.85055633	6.37006413	-1.282952695
C	17.82892411	5.873409519	-0.544062194
H	17.74429651	6.468739716	0.382719009
H	18.40309734	4.968012382	-0.277893761
C	18.61669582	6.682087058	-1.592475216
H	18.70710526	6.085602596	-2.516844226
H	18.03924253	7.583259133	-1.862133975
C	20.01843613	7.097293552	-1.112321253
H	20.6310729	6.215505092	-0.870421048
H	20.55026572	7.674599223	-1.882238966
C	0.384181281	14.09870018	10.14632583
H	1.148470804	14.13851502	9.349821985
H	-0.53928223	14.5208216	9.710970366
C	0.835291589	14.97955611	11.32687987
H	0.07068993	14.93724982	12.12287611
H	1.758937735	14.55803726	11.76233705
C	1.083086256	16.44935334	10.93951846
H	1.849982106	16.49174844	10.14561086
H	0.160278498	16.86984037	10.50127068
C	1.528381428	17.3304656	12.12207187
H	2.451519812	16.91041345	12.5599136
H	0.761646356	17.28634237	12.91584341
C	1.775047184	18.80093055	11.73595983
H	2.54386439	18.84450917	10.94393518
H	0.852785717	19.22020863	11.29528261
C	2.21603724	19.68425578	12.91820951
H	1.447417829	19.6415424	13.71061903
H	3.139080958	19.26731206	13.35982138
C	2.460999548	21.15446826	12.53018698
H	3.229769485	21.19640445	11.7391048
H	1.53881053	21.56950346	12.08720653
C	2.898362977	22.03390605	13.7149871
H	3.835689435	21.6639582	14.15755265
H	3.063152936	23.07457428	13.40090628
H	2.133965433	22.03920435	14.50721845

6. References

(1) Lucassen-Reynders, E. H.; Cagna, A.; Lucassen, J. Gibbs Elasticity, Surface Dilational Modulus and Diffusional Relaxation in Nonionic Surfactant Monolayers. *Colloids Surfaces A Physicochem. Eng. Asp.* **2001**, *186* (1–2), 63–72. https://doi.org/10.1016/S0927-7757(01)00483-6.

(2) *Interfacial Rheology*, 1st ed.; Miller, R., Liggieri, L., Eds.; CRC Press: Leiden Boston, 2009.

(3) Lucassen-Reynders, E. H.; Lucassen, J.; Garret, P. R.; Giles, D.; Hollway, F. Dynamic Surface Measurements as a Tool to Obtain Equation-of-State Data for Soluble Monolayers. In *Adv Chem Ser*; 1975; pp 272–285. https://doi.org/10.1021/ba-1975-0144.ch021.
